# Pulmonary papillary adenoma presenting in central portion: a case report

**DOI:** 10.1186/s13000-015-0425-7

**Published:** 2015-10-17

**Authors:** Xu-Yong Lin, Qiang Han, En-Hua Wang, Yong Zhang

**Affiliations:** Department of Pathology, the First Affiliated Hospital and College of Basic Medical Sciences, China Medical University, Shenyang, 110001 China; Institute of Pathology and Pathophysiology, China Medical University, Shenyang, 110001 China

**Keywords:** Pulmonary papillary adenoma, Lung tumor

## Abstract

Pulmonary papillary adenoma is a very rare tumor usually presenting in periphery of the lung. Herein, we present a case of pulmonary papillary adenoma located in central portion of the lung in a 17 year-old Chinese female. A well-defined mass was incidentally detected at right pulmonary hilar region by imaging examination. Histologically, the tumor is predominantly composed of abundant papillary structures lined by columnar to cuboidal epithelial cells resembling type II pneumocytes. Immunohistochemical staining showed that the epithelial cells were diffusely positive for cytokeratin, cytokeratin7, TTF-1, EMA, surfactant apoprotein A, Napsin A, P63 and β-catenin. The Ki-67 proliferation index was approximately 2 %. Based on morphologic features and the immunohistochemical profile, the tumor was consistent with pulmonary papillary adenoma. Thus, it should be noted that pulmonary papillary adenoma was also a possible diagnosis for a central mass.

## Background

Pulmonary papillary adenoma is a very rare tumor that first described by Fantone et al. in 1982 [1]. So far, less than 25 cases were reported in the English literature [2–16]. The reported cases predominatly occurred in periphery of the lung. In contrast, we present a case of pulmonary papillary adenoma located in the central portion of the lung in a 17 year-old Chinese female. This tumor was generally considered benign; however, some scientists thought that it might have malignant potential because of its microinvasive characteristics [9, 10]. The patient was alive with no evidence of tumor recurrence or metastasis within 12 months of follow-up.

## Case presentation

### Clinical history

**Fig. 1:**
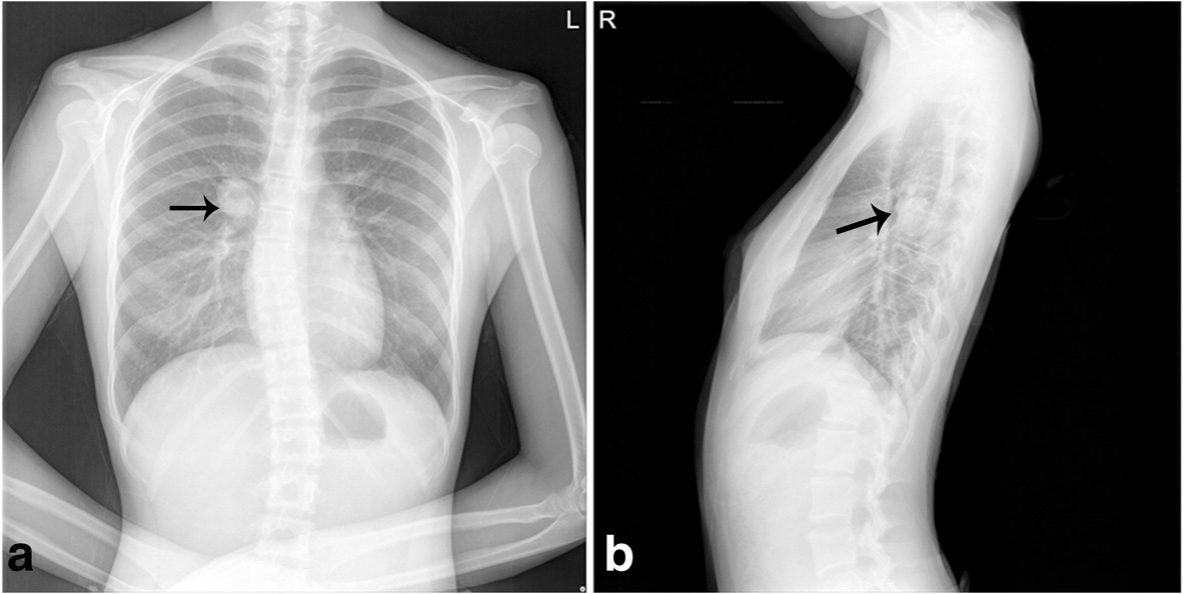
The X-ray manifestation of the tumor. **ab**, The tumor was relatively well circumscribed, located in the hilar region of the right lung

A 17-year-old female without a history of smoking was admitted to our hospital for complaining of a right pulmonary nodule incidentally detected during routine examination. The patient was asymptomatic; physical examination and routine laboratory studies were all within normal values. X-ray demonstrated that there was a well-defined solid mass measuring 3.13 cm in the diameter at the right pulmonary hilar region (Fig.1). In the current visit, the patient underwent wedge resection in our hospital. The postoperative course was uneventful, and there was no evidence of disease 12 months later.

## Materials and methods

The resected specimens were fixed with 10 % neutral-buffered formalin and embedded in paraffin blocks. Tissue blocks were cut into 4-μm slides, deparaffinized in xylene, rehydrated with graded alcohols, and immunostained with the following antibodies: cytokeratin (CK), cytokeratin7(CK7), CD68, Vimentin, thyroid transcription factor 1 (TTF-1), epithelial membrane antigen (EMA), surfactant apoprotein A (SPA), Napsin A, synaptohysin, CD56, P63 and β-catenin, p53 and Ki-67. Sections were stained with a streptavidin-peroxidase system (KIT-9720, Ultrasensitive TM S-P, MaiXin, China). The chromogen used was diaminobenzidine tetrahydrochloride substrate (DAB kit, MaiXin, China), slightly counterstained with hematoxylin, dehydrated and mounted. For the negative controls, the primary antibody was replaced with PBS. This study was prospectively performed and approved by the institutional Ethics Committees of China Medical University and conducted in accordance with the ethical guidelines of the Declaration of Helsinki.

## Results

### Gross features

**Fig. 2:**
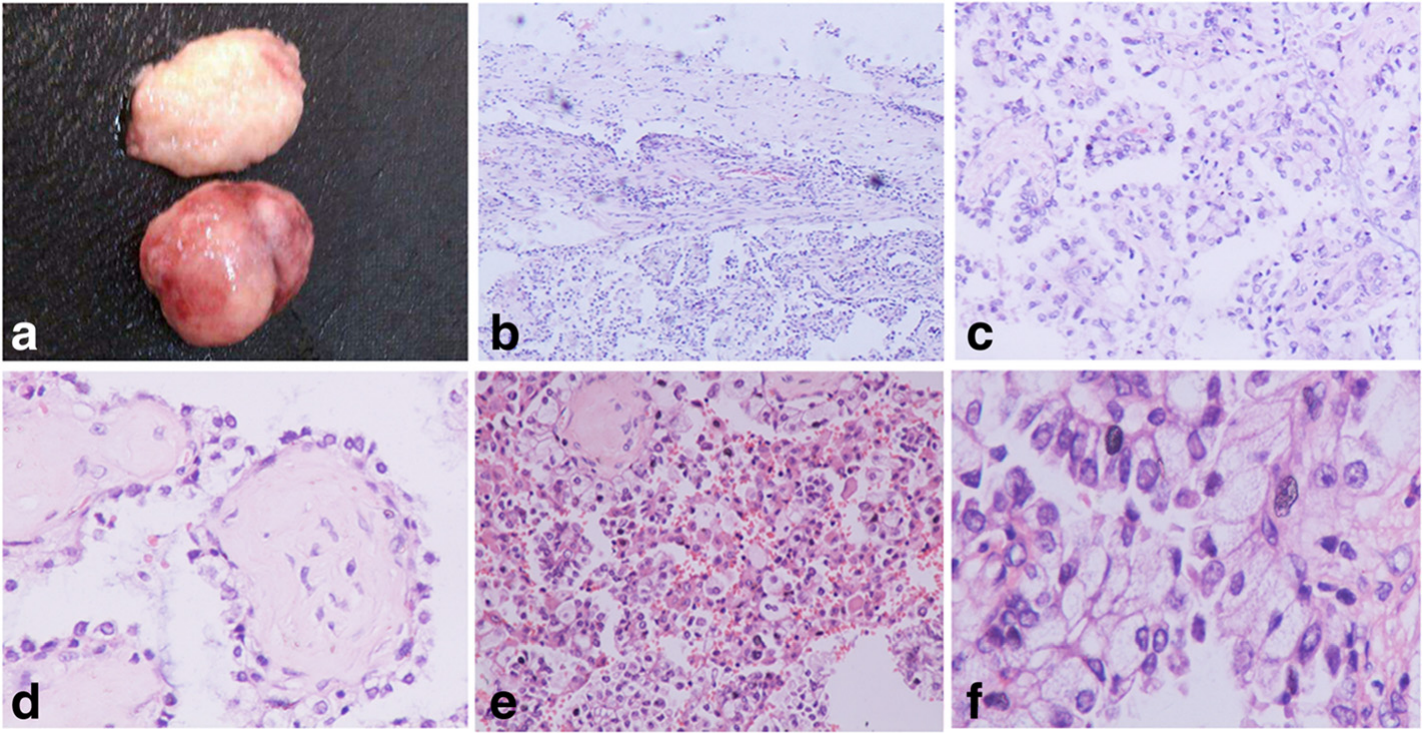
Morphological change of the tumor. **a**, Grossly, the mass was well circumscribed, with a firm and grey-white cut face. **b**, The tumor was encapusuled by the fibrous capsule. **c**, The tumor was composed predominantly of papillary structures with fibrovascular cores. **d**, The abundant hyalinized collagen with few cells was focally present in the core of the papillary structure. **e**, Numerous histiocytes was present in the outer spaces of papillary structures, which might cause a diagnostic confusion. **f**, The lining cells were columnar or cuboidal with mild atypia and extremely rare mitosis, and numerous nuclear inclusions

Grossly, the mass was approximately 3.0 × 2.9 × 2.6 cm, and was relatively well circumscribed. The cut face was firm and grey-white or grey-yellow in color (Fig.2a).

### Histologic features

Histologically, the tumor was relatively well defined, and there was a fibrous capsule around the tumor. The capsule infiltration, normal lung tissue, vessels and pleura invasion was not present in the tumor (Fig.2b). The tumor was predominantly composed of papillary structures with fibrovascular cores (Fig.2c). Focally, the abundant hyalinized collagen with few cells was present in the core of the papillary structure reminiscent of sclerostic pattern of sclerosing pneumocytoma. Contrastly, the stroma of papillary structure lacked the polygnonal cells presenting in sclerosing pneumocytoma (Fig.2d).

The lining cells on the papillary pattern were columnar or cuboidal with mild atypia, clear cytoplasm, fine chromatin and inconspicuous or small nucleoli. The numerous nuclear inclusions were present in the epithelial cells. The mitosis of the cells is very rare (Fig.2f). However, focally, the cells showed florid hyperplasia, and form the micropapillary or irregular cribriform pattern, which might pose a diagnostic challenge. In addition, abundant histiocytes were present in the spaces of the tumor (Fig.2e).

### Immunohistochemical staining and molecular detection

**Fig. 3:**
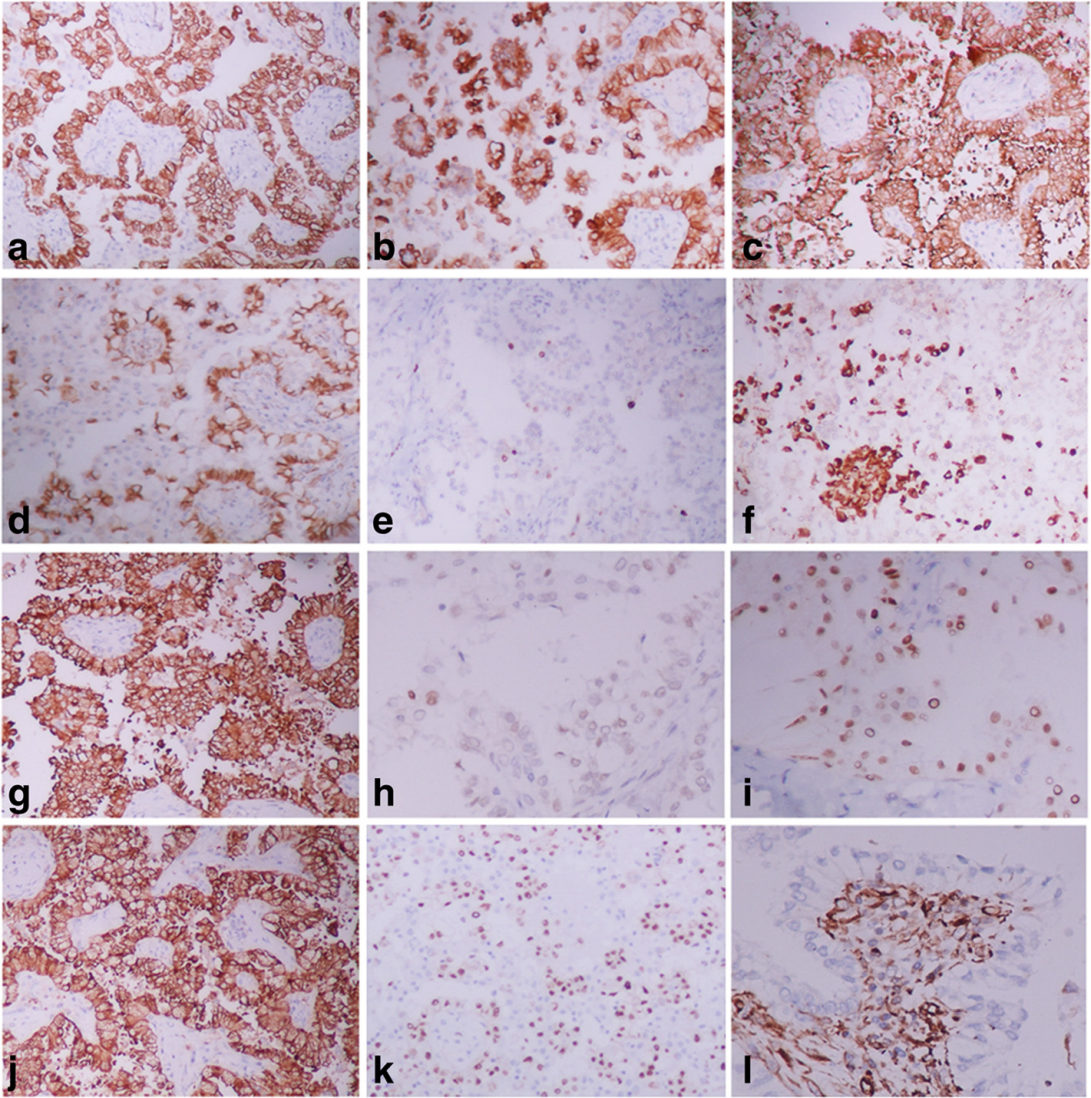
Immunohistochemical staining of the tumor. **a**, **b**, **c**, The lining cells were strongly positive for CK, CK7 and EMA respectively. **d**, The constant and strong membranous staining for β-catenin was seen in the tumor cells. **e**, Ki-67 proliferative index was less than 2 %. **f**, The CD68 staining highlighted the presence of histiocytes. **g**, the cells were diffusely positive for SPA. **h**, Scattered cells showed a weak staining for P53. **i**, the cells were also diffusely positive for P63. **j**, Napsin A was strongly and diffusely expressed in the tumor cells. **k**, TTF-1was positively expressed in the lining cells in contrast to the negative expression in the cells of the stroma. **l**, Vimentin was expressed in the stroma rather than the lining cells

Immunohistochemical staining showed that the lining cells were diffusely positive for TTF-1, EMA, CK, CK7, SPA, P63 and Napsin A, and negative for vimentin, synaptophysin and CD56. The epithelial cells also showed a strong membranous staining for β-catenin. In addition, the cells of the stroma were negative for TTF-1, β-catenin and EMA, indicating the lack of epithelial differentiation. CD68 staining highlighted the presence of histiocytes in the tumor. Few cells showed a weak P53 staining. Ki-67 was expressed in less than 2 % of all tumor cells (Fig. 3). According to the morphological and immunohistochemical findings, the tumor was consistent with papillary adenoma.

We then examined the EGFR and K-ras gene mutations, and failed to find the mutations in this tumor.

## Discussion

Pulmonary papillary adenoma is a very rare tumor. So far, fewer than 25 cases were reported in the English literature [1–16]. In 1980, Spencer et al. reviewed 19 cases of papillary, non-invasive tumors arising from the bronchial epithelium, of which two cases were described as Clara cell origin [2]. In 1982, Fantone et al. used the term papillary adenoma to describe the tumor showing type 2 pneumocytes or Clara cells differentiation, as the presence of cytoplasmic dense granules and whorled lamellar membrane inclusions in the cells [1]. Subsequently, approximately 20 cases were reported [3–16]. Of them, the majority showed type 2 pneumocytes differentiation. Thus, papillary adenoma was believed to be derived from primitive mutipotential respiratory epithelium with bidirectional differentiation [3, 7–10].

Histologically, the tumor comprised by the papillary structures with fibrovascular stroma. The lining cells were usually single layer, cuboidal to columnar. In our case, the cells focally showed florid hyperplasia, formed the micropapillary, sheets or irregular cribriform pattern. This might post a great challenge, especially during the frozen section diagnosis. In addition, it was noted that the numerous nuclear inclusions were present in the epithelial cells, which was commonly present in tumors showing type 2 pneumocytes differentiation [17, 18].

If the tumor showed type 2 pneumocytes differentiation, the cells ultrastructurally contained lamellar bodies and positive for surfactant apoprotein antigen; where staining positively for a Clara cell-specific antigen indicated the differentiation towards Clara cell. The present case was diffusely positive for SPA, indicating the tumor mainly differentiated towards type 2 pneumocytes. Immunohistochemically, the neoplastic cells also showed reactivity for TTF-1, EMA, CK and CK7 [15]. In addition to the above markers, our case was also positive for Napsin A, a useful marker for type II pneumocytes [19]. Surprisingly, in the present case, the cells also demonstrated diffusely reactivity for P63. P63 was mainly expressed in squamous cells, rarely expressed in pneumocytes. It is unclear the significance of diffuse P63 expression in our case.

According to the literature review from Kristine et al., papillary adenoma predominatly occurred in periphery of the lung [15]. In the contrast, our present case was located in the hilar portion of the lung. Although papillary adenoma was thought as a peripheral lesion, it should be noted that this tumor maybe a diagnosis as to the mass located in the hilar area.

It was still debated about the clinical behavior of this tumor. The neoplastic cells were usually lacked marked atypia, high mitosis rate and Ki-67 proliferation rate. Thus, it was generally considered benign; however, some authors advocated that it might have malignant potential because of the infiltration into the capsule, into the adjacent lung parenchyma or visceral pleura. In our case, the tumor was relatively well encapsuled and there was no evidence of capsule, normal lung tissue, vessels and pleura invasion. The patient was alive with no evidence of tumor recurrence or metastasis within 12 months of follow-up. Nevertheless, the longer follow–up was still necessary, as the potential relationship between papillary adenoma and adenocarcinoma [9].

The differential diagnosis of the tumor includes pulmonary sclerosing pneumocytoma, alveolar adenoma, atypical adenomatous hyperplasia, and papillary adenocarcinoma. Pulmonary sclerosing pneumocytoma is a relatively rare but distinctive tumor [20, 21]. The immunohistochemical and molecular findings have strongly documented that pulmonary sclerosing pneumocytoma also originates from primitive respiratory epithelium [22]. Thus, pulmonary sclerosing pneumocytoma may have the same origin with papillary adenoma. It is characterized by two cell types, namely surface cuboidal cells and polygonal cells located in the stroma of the papillary structures. The presence of polygonal cells in pulmonary sclerosing pneumocytoma, which also showed positivity for TTF-1, was a peculiar feature for differential diagnosis. Additionally, in the present case, the cells showed strong and constant membranous staining for β-catenin, which was consistent with the staining pattern in cuboidal cells of pulmonary sclerosing pneumocytoma, indicating their common origin [21].

Alveolar adenoma is characterized by multiple cystic spaces lined by single layer of flat to cuboidal to type II pneumocytes containing a granular material. The cysts are often filled with an eosinophilic proteinaceous fluid. The widespread presence of papillary morphology could aid in excluding this tumor. Atypical adenomatous hyperplasia is a small (usually ≤0.5 cm) preinvasive lesion. It is characterized by proliferation of mildly to moderately type II pneumocytes with lepidic pattern along the alveolar walls. The presence of broad papillary structures could also distinguish the papillary adenoma from atypical adenomatous hyperplasia. Moreover, the lack of cellular atypia and mitosis could exclude papillary adenocarcinoma. The gene mutations of EGFR and K-ras could be involved in pulmonary adenocarcinoma, which was well documented [23]. The absence of EGFR and K-ras gene mutations in papillary adenoma might also be helpful for differential diagnosis.

## Conclusion

Pulmonary papillary adenoma is an extremely rare tumor characterized by widespread papillary structures. The reported cases predominatly occurred at the periphery, nevertheless, we presented the case located in central portion of the lung, indicating it should still be considered for a central mass.

## Consent

Written informed consent was obtained from the patient for publication of this case report and accompanying images. A copy of the written consent is available for review by the Editor-in Chief of this Journal.
